# Stress “deafness” in a Language with Fixed Word Stress: An ERP Study on Polish

**DOI:** 10.3389/fpsyg.2012.00439

**Published:** 2012-11-01

**Authors:** Ulrike Domahs, Johannes Knaus, Paula Orzechowska, Richard Wiese

**Affiliations:** ^1^Philipps-Universität MarburgMarburg, Germany; ^2^University of PotsdamPotsdam, Germany; ^3^Adam Mickiewicz UniversityPoznań, Poland

**Keywords:** stress “deafness,” fixed stress system, prosodic representation, P300, generalized error-detection mechanism

## Abstract

The aim of the present contribution was to examine the factors influencing the prosodic processing in a language with predictable word stress. For Polish, a language with fixed penultimate stress but several well-defined exceptions, difficulties in the processing and representation of prosodic information have been reported (e.g., Peperkamp and Dupoux, 2002). The present study utilized event-related potentials (ERPs) to investigate the factors influencing prosodic processing in Polish. These factors are (i) the predictability of stress and (ii) the prosodic structure in terms of metrical feet. Polish native speakers were presented with correctly and incorrectly stressed Polish words and instructed to judge the correctness of the perceived stress patterns. For some stress violations, an early negativity was found which was interpreted as a reflection of an error-detection mechanism. In addition, exceptional stress patterns (=antepenultimate stress) and post-lexical (=initial) stress evoked a task-related positivity effect (P300) whose amplitude and latency is correlated with the degree of anomaly and deviation from an expectation. In contrast, violations involving the default (=penultimate stress) did not produce such an effect. This asymmetrical result is interpreted to reflect that Polish native speakers are less sensitive to the default pattern than to the exceptional or post-lexical patterns. Behavioral results are orthogonal to the electrophysiological results showing that Polish speakers had difficulties to reject any kind of stress violation. Thus, on a meta-linguistic level Polish speakers appeared to be stress-“deaf” for any kind of stress manipulation, whereas the neural reactions differentiate between the default and lexicalized patterns.

## Introduction

A major step in recognizing a word, more generally a lexical item, consists in the access to its phonological form. This phonological form minimally contains the necessary information making the item distinct from other lexical items. In classical phonology, the sequence of phonemes constituted this minimal information. However, it is also obvious that prosodic, i.e., non-segmental, information may often be part of the phonological form used in lexical access. This is obvious for lexical tone features (as in Chinese) or tone accent (as in Japanese), and also for lexical stress (as for instance Spanish; Navarro Tomas, 1946), in which stress positions are not predictable on the basis of a restricted set of stress rules.

One factor affecting the role of stress in processing is the type of stress system involved. While stress may serve to distinguish lexical items (e.g., in Spanish or even more so in Russian), other languages such as Polish show a more or less fixed stress pattern. More precisely, Polish has a default pattern with a high degree of predictability, but also some exceptions to this default or regular pattern, as will be discussed in the section on Polish word stress below.

Recently, a number of experimental studies have investigated the role of stress and of stress-related prosodic units in word recognition. A crucial finding of these studies is that speakers of different languages may show signs of stress “deafness,” a general insensitivity to word stress properties, depending on the status of stress in their language. Stress “deaf” L2 learners have difficulties to represent distinctive stress information because suprasegmental information is not relevant for words of their language. According to crosslinguistic studies by Peperkamp and Dupoux (2002) and Peperkamp et al. (2010), native speakers of Polish show only an intermediate level of stress “deafness” due to the existence of a small group of exceptions from predictable stress.

Another issue of word stress that will be investigated in the present study is that of prosodic structures involved in stress representation and stress processing. In current phonological theory and description (as developed, e.g., by Liberman and Prince, 1977; Hayes, 1995), stress is interpreted as a property of prosodic units such as the foot (F) or the prosodic word (ω), expressing a strong-weak relation between syllables and feet {e.g., [(kin_σs_der_σw_)_Fs_(gar_σs_ten_σw_)_Fw_]_ω_}. In studies utilizing event-related potentials (ERPs) on German word stress processing, Knaus et al. (2007) and Domahs et al. (2008) found that participants, when confronted with correctly and incorrectly stressed words, perceive differences between stress shifts to a head syllable of a foot or a weak syllable of a foot. The latter violation type was less expected and constituted a stronger violation from the expected stress pattern. These studies show that the processing of incorrectly stressed words can be used to identify prosodic structures of stress systems, in particular whether syllables are organized into feet and which type of feet is constructed. With respect to Polish, it has been suggested that bisyllabic trochees are built from right to left within prosodic words (Dogil and Williams, 1999), but it is under debate whether feet play a role in the Polish metrical system at all. While non-primary feet within words are often the landing site for secondary stresses, no phonetic cue has been identified to be correlated with secondary stresses. Therefore, phonetic analyses alone cannot answer the question whether secondary stresses exist.

In the present paper, the aim is to investigate which factors influence the processing of word stress in Polish. To this end, the following questions are raised:

(a) To what extent are Polish speakers stress “deaf” given the existence of an exceptional pattern? Does the sensitivity to stress depend on the distinction between default and non-default stress?
(b) Are foot structures and secondary stresses part of the Polish word stress system?

To address these questions, we performed an ERP study on Polish stress perception, using the stress evaluation paradigm as has been introduced in previous studies on word stress perception (e.g., Knaus et al., 2007; Domahs et al., 2008, 2012).

### Studies on stress “deafness”

A number of studies (e.g., Dupoux et al., 1997, 2001; Peperkamp and Dupoux, 2002) discussed how speakers of languages with fixed stress (Hungarian, Finnish) use stress information in language processing. In sequence recall tasks, participants were asked to memorize sequences of nonce words, which were minimal pairs differing either in a phonemic contrast or in stress.

The study of Peperkamp and Dupoux (2002) suggests that the degree of predictability of stress positions in a word is linked to the degree of sensitivity to stress variation and, in turn, the capacity to process stress information at an abstract level. Their main argument is that native speakers of a language with a predictable stress pattern do not need to have stress information stored in the phonological representation, which results in difficulties with memorizing stress contrasts in L2 learning. Having studied four European languages (Finnish, French, Hungarian, Polish) with non-contrastive stress, Peperkamp and Dupoux (2002) propose a typology of stress deafness. They postulate that the size of the deafness effect corresponds to the degree of regularity of stress patterns in a language and, as a result, the ease with which stress can be acquired by infants. Native speakers of a language with variable stress pay attention to stress information, since this information allows to differentiate between lexical items. When the distribution of stressed syllables is predictable, stress information is ignored as it has no contrastive function. Peperkamp et al. (2010) showed that out of several examined languages with fixed stress, speakers of Standard and Southeastern French, Finnish as well as Hungarian exhibit strong stress deafness, as opposed to stress-sensitive speakers of Spanish, a language with a non-predictable stress pattern.

Schmidt-Kassow et al. (2011b) performed ERP-studies with French participants learning German, and found that French native speakers with a high proficiency in German as L2 are insensitive to metrical violations of a homogeneous trochaic pattern in sentences (e.g., ′*Vera ′hätte ′Christoph ′gestern ′morgen* *du′ZEN ′können. Schmidt-Kassow et al., 2011b: p. 3). This is shown by the lack of a P600 effect which is usually evoked by such metrical violations. However, confronted with metrical violations in general grouping strategies of tones, French participants were able to perceive strong-weak patterns. The perception of deviations to this pattern evoked a positivity effect. The authors conclude that the difficulties to perceive trochaic patterns for French native speakers are specific for language processing and related to the syllable-timed word processing of French in contrast to the trochaic system of German.

On the other side, speakers of languages with predictable stress but few exceptions show different results than French speakers. Peperkamp et al. (2010) classified native speakers of Polish as belonging to an intermediate group with weak stress “deafness” (according to the authors about 0.1% of words exhibit exceptional stress in Polish). Speakers of Polish were better at recalling sequences of differently stressed words than speakers of the remaining languages with predictable stress and no exceptions and performed worse than speakers of Spanish, which has unpredictable stress and a large number of lexical exceptions (17%). Therefore, the small number of exceptions from the predictable stress pattern influences the ability to represent stress information at an abstract level significantly. The question addressed in the present paper is whether the exceptions influence the perception of stress positions generally or whether this might be the case only for the exceptional patterns. In this respect, results from an ERP study on Turkish are of particular interest.

Turkish is another example of a language with a largely fixed stress assignment: it displays default stress on the final syllable with well-defined exceptions from this pattern. Domahs et al. (2012) conducted an EEG study on the processing of word stress in Turkish, showing that default final stress is processed differently from non-default stress on the penult or antepenult. Incorrect final stress led to an N400-like effect, while incorrect penultimate and antepenultimate stress caused a positivity effect that was interpreted to belong to the P3b family. These results demonstrate that Turkish speakers are sensitive to the lexicalized stress patterns but not to the default stress.

In the present paper, it will be examined whether word stress processing in Polish depends on the default/non-default distinction as well. In contrast to Turkish, Polish words with exceptional stress allow for regularization. Therefore, we expect the asymmetry between default and non-default patterns to be less pronounced.

### Studies on metrical structure

Domahs et al. (2008) studied the perception of word stress in German trisyllabic words representing three different stress patterns: antepenultimate (e.g., ′*Lexikon* “lexicon”), penultimate (*Ka*′*sino* “casino”), and final (*Vita*′*min* “vitamin”). All words were presented auditorily, once with the correct stress pattern and once with each of the two incorrect ones. The participants’ task was to decide whether stress was assigned to the appropriate syllable or not by pressing either a “yes” or a “no” response button. Before the participants heard the auditory stimuli, the target words were presented visually on a computer screen to avoid a lexical search effect and to build up an expectation for the correct stress pattern, which can be violated more or less by different violation types.

The ERP responses to words in correct and incorrect conditions showed that stress patterns resulting in the change of the word’s metrical structure produced a positive deflection, while stress violations maintaining the original foot structure did not. This positivity effect was interpreted to represent an instance of the P3b family (e.g., Picton, 1992; Coulson et al., 1998) reflecting the degree of abnormality from an expected (=correct) form. This component only occurs if the participants’ attention is directed toward the metrical manipulation by the given task. Moreover, this experiment demonstrated the importance of binary foot structure as a crucial part of the German word-prosodic system. The time course of the effects shows that stress information is processed as soon as information about prominence relations between the first two incoming syllables of a word becomes available. Overall, this method enables to identify syllables that are potentially stressable in a certain stress system, i.e., head syllables of feet.

With respect to Polish, the present study investigates whether foot structure plays a similar role in Polish stress perception. If it does, stress shifts to head syllables of feet should be perceived as less deviant from the expected pattern than shifts to weak syllables of feet.

### Polish word stress

Polish is a language with a fixed stress system with primary stress on the penultimate syllable as a default (Wierzchowska, 1971; Comrie, 1976; Hayes, 1995), irrespective of the morphological composition of a word. Word stress is, thus, predictable on the basis of word boundaries, and phonological and morphological factors do not contribute to stress assignment. Stress moves along with additional prefixes and suffixes, which results in penultimate stress in the majority of phonological words, as shown in (1).

**Table:** 

(1)	′ję.zyk	“language [nominative singular]”
	ję.zy.′ka.mi	“language [instrumental plural]”
	ję.zy.ko.′znaw.ca	“linguist [nominative singular]”
	ję.zy.ko.znaw.′ca.mi	“linguist [instrumental plural]”
	ję.′zy.czny	“lingual [masculine singular]”

The Polish stress rule, however, has some well-defined groups of exceptions in which stress is assigned not to the penult, but to the antepenultimate syllable. These subgroups include mainly borrowings, in which stress on the antepenultimate syllable is the canonical form. In addition, antepenultimate stress applies to the first and second person plural forms of past tense verbs, as in *czy*.′*ta.lis.my* “we were reading (masculine).” Stress does not move rightward with the attachment of {-smy} and {-scie} to the stem, as with some other suffixes. Compare the stress-neutral suffixes given in (2) to stress-sensitive suffixes presented in (3). Attaching the former suffixes does not affect stress placement, so that stress surfaces on the antepenultimate syllable, while the suffixation of the latter causes a change in stress position.

**Table:** 

(2)	Stress fixed on the stem with stress-neutral suffixes
	a. u.′czy.li “learn [3rd plural past tense]”
	b. u.′czy.li.śmy “learn [1st plural past tense]”
	c. u.′czy.li.ście “learn [2nd plural past tense]”
(3)	Stress shifted to the penult with stress-sensitive suffixes
	a. {-cja}: pro.′duk.cja “production”, pro.duk.′cja.mi “production [instrumental plural]”
	b. {-ość}: ′czyn.ność “activity”, czyn.noś.′cio.wy “functional”
	c. {-źń}: ′przy.jaźń “friendship”, przy.′jaź.ńić (się) “befriend”

As regards loans from Greek and Latin, all words ending in {-ika} and {-yka} are stressed on the antepenult, e.g., *a′kustyka, gra′matyka, ′klinika*. Although this stress pattern is considered to be the preferred one in normative accounts (Dlubisz, 2006), Polish speakers tend to shift it to the penult (Bajerowa, 2001; Nagórko, 2006). Therefore, words with stress on the antepenultimate syllable, such as *ma.te*.′*ma.ty.ka* “mathematics,” ′*pre.zy.dent* “president,” and ′*sta.tu.a* “statue” have an alternative penultimate stress pattern available, which, in turn, points to the preference for regularization of exceptions. Variation of stress is also found in proper nouns (e.g., ′*Waszyngton* is often realized as *Wa*′*szyngton*) and in past tense verbs in colloquial speech (Bajerowa, 2001; Nagórko, 2006). Apart from the exceptions with antepenultimate that are relevant for the purpose of the present paper, further exceptions with final and pre-antepenultimate stress also exist.

In addition to penultimate and exceptional stress, primary stress in Polish can also be assigned to the initial syllable of a word. This pattern occurs primarily in an emphatic context (Dłuska, 1974) and can be classified as a kind of post-lexical stress. Moreover, there is a strong tendency for secondary stress to be initial (e.g., _′_*au.to.bu*.′*so.wy* “bus”_adj_). Work by Dogil and Williams (1999) shows that the primary penultimate stress and the secondary initial stress are closely related, i.e., in narrow focus the prominence relations between the two patterns can be switched as in ′*mar.ma.la*._′_*do.wy* instead of _′_*mar.ma.la*.′*do.wy* “marmalade-like.” This shift, however, is claimed to apply only to words composed of at least four syllables. Wierzchowska (1971; pp. 219–221) also states that the reversed stress parameter, i.e., primary initial and secondary penultimate, is the preferred accentual norm for longer words in the spontaneous speech of the young urban population.

With respect to the phonetic implementation of word stress in Polish, it displays particular phonetic reflexes irrespective of stress position or syllable weight. Łukaszewicz and Rozborski (2008) found the following order of importance for phonetic cues to lexical stress in Polish: intensity > fundamental frequency > duration. This result suggests that vowel length plays no role in stress assignment.

On the whole, Polish is a language with primary word stress normally fixed on the penultimate syllable, but with a range of well-defined exceptions, and presumably with secondary stress on the initial syllable (for an overview of stress patterns in Polish see Table 1). In the present experiment, we will not only examine the role of word stress for the processing of Polish words but also address the question of the existence of syllabic trochees. Are words like *witamina* “vitamin” structured prosodically as [(_′_*wi ta*)_Fw_ (′*mi na*)_Fs_]_ω_?

**Table 1:** **Overview of stress patterns in Polish**.

Stress pattern	Context	Example
PU	Majority of native vocabulary	*Ję.zy.ko*.′*znaw.ca*	“linguist”
	Nativized borrowings	*Le*. ′*ber.ka*	“liverwurst”
IN	Emphasis	′*Ma.te.ma.ty.ka*	“mathematics”
	Strong secondary stress	′*Au.to.bu*._′_*so.wy*	“bus-like”
	Non-nativized borrowings	′*Hu.ckle.be.rry*, ′*Ei.sen.ho.wer*	
APU	First and second person plural past verbs	*Czy*.′*ta.liś.my*	“we were reading”
	Singular and third person plural conditional verbs	′*Zro.bił.bym*	“I would have done”
	Numbers with {-kroc}, {-sta}, and {-set}	′*Ty.siąc.kroć*	“one thousand times”
	Several Polish words (optionally)	′*Sta.tu.a*	“statue”
	Loan words ending in {-ika}/{-yka}	*A*.′*ku.sty.ka*	“acoustics”
	Loan words of Latin and Greek origin	*U.ni*. ′*wer.sy.te*	“university”

*PU stands for penultimate stress, APU for antepenultimate stress, and IN for initial stress*.

### Hypotheses

The present research questions address the role of stress predictability and the influence of prosodic structure for the processing of Polish words. In general, we are interested in the status of penultimate, antepenultimate, and initial stress in online processing (in comparison to the results obtained for Turkish, Domahs et al., 2012) and in finding evidence for foot structure in Polish.

The first set of hypotheses is based on the assumption that the lexical distribution of word stress in Polish is a crucial factor in determining the EEG responses (following Domahs et al., 2012). Given that penultimate stress is the default stress pattern for Polish words, stress shifts toward the default position should not evoke a late positive component while stress shifts away from the default (to the antepenultimate) should. In a similar experiment on Turkish, it was found that Turkish participants had selective difficulties to judge stress shifts involving the default stress and as a result no positivity effect occurred.

The second set of hypotheses is derived from the premise that the prosodic structure of words plays an important role in their processing (following Domahs et al., 2008). The responses to stress shifts should be mostly determined by the syllabic trochees suggested for Polish. Quadrisyllabic words bearing penultimate stress are expected to have the surface foot structure (_′_σ σ)(′σ σ) by default while words bearing antepenultimate stress are expected to have the lexically specified foot structure σ (′σ σ) σ. What follows from these assumptions about metrical structures is that any shift of stress will cause a restructuring of feet, except for a shift from the penultimate to the initial syllable. That is, a shift from *(*_′_*wi ta*)(′*mi na)* “vitamin” to *(*′*wi ta*)(_′_*mi na)* keeps the foot structure intact. In Domahs et al. (2008) it was found for German that stress shifts that maintained the foot structure did not cause a late positive component.

## Materials and Methods

### Participants

Thirty right-handed native speakers of Polish (16 women) living in the region of Wielkopolska participated in the study. Each participant had normal or corrected-to-normal vision and no hearing deficit. Their mean age was 23, ranging from 19 to 31 years. No subject reported to have been brought up in a bilingual or multilingual context. Four participants (three women) were excluded from the final data analysis due to excessive eye-movement artifacts. Each participant was paid for her/his contribution. EEG recordings took place in the Center for Speech and Language Processing in the Faculty of English at Adam Mickiewicz University, Poznan.

### Material

The participants were presented with quadrisyllabic Polish words (see Appendix for the complete list) with canonical penultimate or antepenultimate stress. For each of these two stress patterns, 15 words were selected. It proved not possible to find a larger number of suitable quadrisyllabic words with antepenultimate stress which are well-known, well integrated, and unambiguous in terms of stress position. Furthermore, words with antepenultimate stress should not predominantly contain the {-ika}/{-yka} suffix. Thus, this set of words consisted of words ending in the {-ika}/{-yka} suffix, a few monomorphemic words, and some proper nouns. In order to increase the number of critical items, each set was presented twice. Word frequency (lemma frequency) of both word sets was controlled by using the full version of the www.korpus.pl, which contains 250 million tokens.

Each word was spoken naturally at a normal speech rate by a native female monolingual speaker of Polish coming from the region of Wielkopolska, recorded with a 16 bit resolution and a sampling rate of 44.1 kHz. The speaker pronounced every word with the correct stress pattern, with incorrect stress on the initial syllable, and incorrect stress on the antepenultimate or penultimate syllable depending on the location of the correct stress. The words were spoken and presented embedded in a carrier sentence. The overall arrangement of stimuli in this experiment is given in (4).

**Table:** 

(4)	Quadrisyllabic stimuli (stress patterns highlighted by capitalization)	
	a.	penultimate stress:
		at.mo.′SFE.ra
		*′AT.mo.sfe.ra
		*at.′MO.sfe.ra
	b.	antepenultimate stress:
		ba.′LI.sty.ka
		*′BA.li.sty.ka
		*ba.li.′STY.ka

The number of critical items presented was 15 types × 2 (penultimate vs. antepenultimate stress) × 3 (correct pattern and two incorrect stress patterns) × 2 (two presentations), resulting in 180 items, i.e., due to the repetition of each word averaging processes were conducted over 30 stimuli per condition. In order to present a balanced number of correct and incorrect items, 30 filler words (presented twice) with correct stress were added to the target nouns. From these filler words, 15 were correctly stressed on the penult and 15 were correctly stressed on the antepenult. The group of filler items consisted of nouns matched to the target nouns in terms of frequency.

All nouns were embedded in the carrier sentence *On powinien powiedzieć* … *wiele razy* “he should say … many times,” in which they were realized in the nominative singular form, with the exception of the plural proper name *Karaiby* “the Caribbean.” The carrier sentence and the particular placement of the critical items in the sentence were chosen to avoid effects of sentence-final lengthening and boundary tones on the critical items. For the preparation of stimuli, each critical item was cut out of the individual carrier sentence and spliced into one identical token of the carrier sentence. Thus, the same token was used for all the target and filler items.

To ensure naturally realized stresses and in particular no difference in the realization of correctly and incorrectly stressed words with the identical stress pattern (e.g., PU correct in *at.mo*. ′*SFE.ra* and incorrect in *ba.li. ′*STY.ka*), a phonetic analysis of the stimuli was conducted for the relevant phonetic parameters of pitch intensity, and duration. These parameters were measured and compared across stimuli with identical stress pattern. As correct initial stress does not occur in the nominal paradigm, quadrisyllabic verbs with correct initial stress were recorded additionally, to allow for a phonetic analysis of initial stress realization. The verbs were recorded for the purpose of phonetic analysis only and were not presented in the experiment. As can be seen from Tables 2 and 3, the realizations of correct and incorrect stress did not differ significantly from each other, i.e., incorrect stress was produced within the same parameter ranges as correct stress.

**Table 2:** **Descriptive statistics of phonetic parameters**.

Stress realized on	Correct stress pattern								
	Initial syllable			Antepenult			Penult		
Initial syllable	*F*0	253.35	(15.35)	*F*0	249.27	(19.11)	*F*0	255.92	(19.30)
	Int.	56.11	(3.72)	Int.	59.29	(3.21)	Int.	56.73	(4.02)
	Dur.	288	(64)	Dur.	218	(77)	Dur.	174	(56)
Antepenult	–			*F*0	243.03	(13.56)	*F*0	244.97	(11.10)
	–			Int.	58.96	(4.32)	Int.	55.85	(5.22)
	–			Dur.	180	(55)	Dur.	237	(75)
Penult	–			*F*0	236.31	(13.40)	*F*0	227.58	(13.25)
	–			Int.	53.96	(6.11)	Int.	58.67	(3.11)
	–			Dur.	242	(48)	Dur.	240	(56)

*Mean values of *F*0 (Hz), intensity (dB), and duration (ms) for the stressed syllable of each stress pattern; correct conditions shaded; SD in parentheses*.

**Table 3:** **Repeated measures ANOVA of each phonetic parameter over the factors correctness (comparison of correct and incorrect stress conditions) and syllables (values of each of the four syllables)**.

Correct initial stress vs. shifted from antepenult		Correct initial stress vs. shifted from penult	
*F*0	*F*(1, 14) = 0.05, *p* > 0.834	*F*0	*F*(1, 14) = 3.21, *p* > 0.095
Int.	*F*(1, 14) = 2.16, *p* > 0.164	Int.	*F*(1, 14) = 0.77, *p* > 0.395
Dur.	*F*(1, 14) = 1.08, *p* > 0.317	Dur.	*F*(1, 14) = 0.85, *p* > 0.371

**Correct antepenultimate stress vs. shifted from penult**		**Correct penultimate stress vs. shifted from antepenult**	

*F*0	*F*(1, 14) = 0.11, *p* > 0.749	*F*0	*F*(1, 14) = 0.02, *p* > 0.877
Int.	*F*(1, 14) = 0.13, *p* > 0.722	Int.	*F*(1, 14) = 1.31, *p* > 0.272
Dur.	*F*(1, 14) = 1.70, *p* > 0.214	Dur.	*F*(1, 14) = 2.64, *p* > 0.127

*Reported are the main effects for the factor correctness*.

### Procedure

For the experimental procedure, we chose a violation paradigm which has proven to be insightful in studies on German word stress (see Knaus et al., 2007; Domahs et al., 2008).

**Figure 1:**
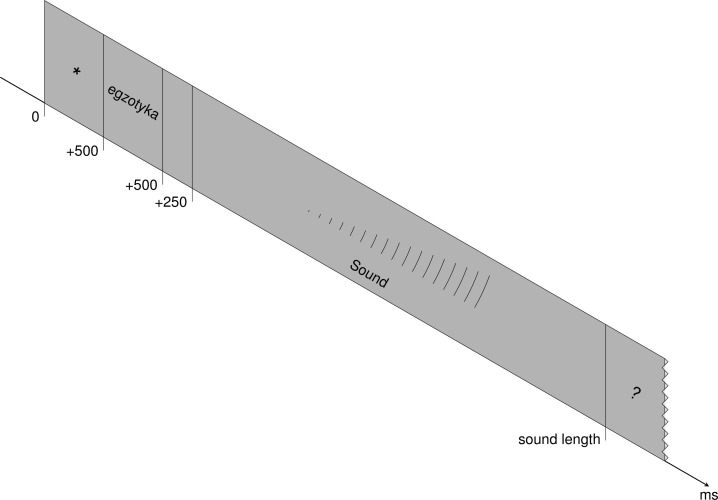
**Trial scheme**.

Participants were seated in front of a screen in a dimly lit and sound-proof room. Each trial started with the visual presentation of a target word on the screen for 500 ms, followed by 250 ms of blank screen. Next, participants were exposed to the same target item in the carrier sentence auditorily (either correct or incorrect). After the presentation of the stimulus, a question mark appeared on the screen with a timeout of 2 s. Participants were instructed to judge the correctness of the stress pattern heard by pressing an appropriate button after the offset of the sentence. To avoid a handedness bias, the assignment of answer keys to yes and no buttons was counterbalanced across participants. After the response, a blank screen appeared before the next trial started. During the period from the offset of the signal to the onset of the next trial, participants were allowed to blink and rest their eyes. Figure 1 depicts the trial sequence used in the present study.

Each participant was exposed to the same set of 240 items in randomized order, divided into six blocks of 40 items each. There were 10 different orderings of blocks, and participants were given the opportunity for a pause after each block. Before the experiment started, they were exposed to a set of 10 practice trials during which they were instructed on the procedure and given feedback if necessary. Running of the experiment took approximately 30 min.

### EEG recordings and analysis

The electroencephalogram (EEG) was recorded by means of 24 Ag-AgCl electrodes with the C2 electrode serving as ground electrode. During the recording, the reference electrode was placed at the left mastoid. EEGs were re-referenced off-line to both mastoids. To control for eye-movement artifacts, vertical eye movements were recorded by electrodes above and below the participants’ left eye, and horizontal eye movements by two electrodes fixed to the outer canthus of both eyes (electrooculogram, EOG). Electrode impedances were kept below 5 kΩ. EEGs and EOGs were amplified using a BrainAmp amplifier (Brain Products, Germany), recorded continuously with a digitization rate of 500 Hz, and filtered off-line with a bandpass filter from 0.3 to 20 Hz.

Trials with eye movements and other types of artifacts with an amplitude above 40 μV were removed from the data set (4.3% of all trials; 3–6% per condition). Furthermore, the data sets of four participants had to be excluded completely from further analysis due to drop-out rates above 33%. Within time windows from word onset up to 1500 ms thereafter, averages were computed over participants, conditions, and electrodes. In the resulting ERPs, negative and positive deflections were analyzed relative to the correct stress condition as a control. For the analysis of ERPs, time windows (see Tables 5 and 6) were selected on the basis of previous studies and by visual inspection of EEG plots, and regions were defined as frontal (F3, Fz, F4), central (C3, Cz, C4), and parietal (P3, Pz, P4). ANOVAs with repeated measures were calculated over the factors stress position (initial, antepenultimate, penultimate) and region (frontal, central, parietal).

## Results

### Behavioral data

**Figure 2:**
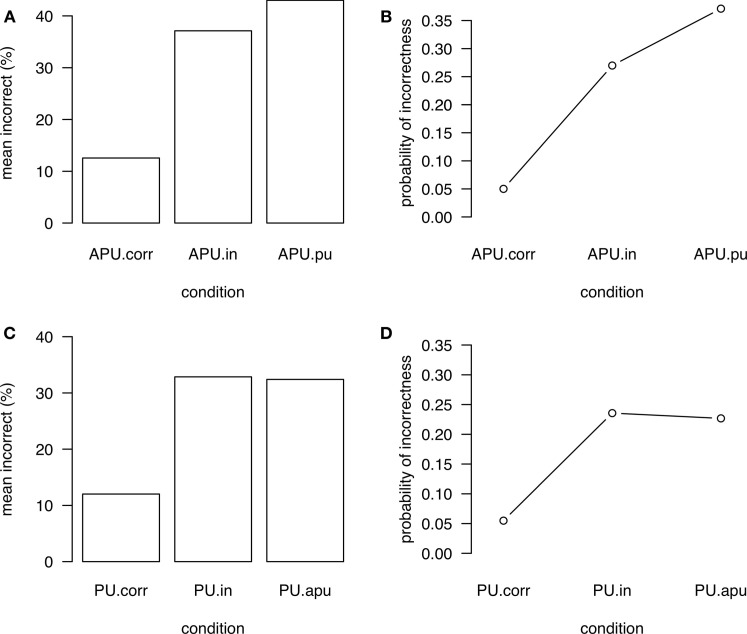
**Descriptive means of incorrect responses (A) for words with canonical antepenultimate stress and (C) for words with canonical penultimate stress and probability of incorrect responses as predicted by the mixed effects regression models (B) for words with canonical antepenultimate stress and (D) for words with canonical penultimate stress**.

For the participants’ judgments of stress patterns, error rates were collected in order to assess the accuracy of stress perception. In Figure 2 (left panels), mean accuracy scores for each stress conditions are illustrated.

For the analysis of the behavioral data we used generalized mixed effects regression models. We devised mixed effects regression models (e.g., Baayen, 2008; Baayen et al., 2008) to test whether the different stress conditions had an effect on the accuracies. Mixed effects regression has the advantage of bringing subject and item variation under statistical control and of being able to deal with unbalanced data sets.

For the mixed effects analysis we used the statistics software R (R Development Core Team, 2011) and the lme4 package (Bates et al., 2011). We fitted generalized mixed effects models for each word type separately (canonical penultimate and canonical antepenultimate stress) with the three conditions per word type as predictors (i.e., “words with canonical penultimate stress, correctly stressed” (PU.corr), “words with canonical penultimate stress, shifted to the initial syllable” (PU.in), “words with canonical penultimate stress, shifted to the antepenultimate syllable” (PU.apu); “words with canonical antepenultimate stress, correctly stressed” (APU.corr), “words with canonical antepenultimate stress, shifted to the initial syllable” (APU.in), “words with canonical antepenultimate stress, shifted to the penult” (APU.pu). The models were fitted over all three conditions per word type and over incorrect conditions per word type (see Table 4). In the resulting four models, accuracy was included as dependent variable. If the participant’s response to a given stimulus was correct this was coded as “corr,” and if not as “incorr.”

**Table 4:** **Mixed effects regression models for accuracies over conditions per word type**.

ACCURACIES IN CONDITIONS OF WORDS WITH CANONICAL ANTEPENULTIMATE STRESS (APU.corr, APU.in, APU.pu)				
**Groups**	**Name**	**Variance**	**SD**	

**Random effects**				
Cond: subject	(Intercept)	1.06776	1.03333	
Item	(Intercept)	0.84451	0.91897	
Subject	(Intercept)	2.42480	1.55718	
Number of obs: 2279, groups: cond:subj, 78; item, 45; subj, 26				

	**Estimate**	**SE**	***z* value**	**Pr(>|*z*|)**

**Fixed effects**				
(Intercept)	−2.9431	0.4710	−6.248	<0.000***
apu.in	1.9480	0.4872	3.998	<0.000***
apu.pu	2.4160	0.4858	4.973	<0.000***
**C**	**AIC**	**BIC**	**logLik**	**Deviance**
0.9052199	1968	2002	−977.9	1956

**ACCURACIES IN INCORRECT CONDITIONS OF WORDS WITH CANONICAL ANTEPENULTIMATE STRESS (APU.in, APU.pu)**				

**Groups**	**Name**	**Variance**	**SD**	

**Random effects**				
Cond:subject	(Intercept)	0.65767	0.81097	
Item	(Intercept)	0.94500	0.97211	
Subject	(Intercept)	3.28243	1.81175	
Number of obs: 1515, groups: cond:subj, 52; item, 30; subj, 26				

	**Estimate**	**SE**	***z* value**	**Pr(>|*z*|)**

**Fixed effects**				
(Intercept)	−1.0111	0.4776	−2.117	0.0343*
apu.pu	0.4825	0.4476	1.078	0.2811
**C**	**AIC**	**BIC**	**logLik**	**Deviance**
0.8981822	1455	1481	−722.3	1445

**ACCURACIES IN CONDITIONS OF WORDS WITH CANONICAL PENULTIMATE STRESS (PU.corr, PU.in, PU.apu)**				

**Groups**	**Name**	**Variance**	**SD**	

**Random effects**				
Cond:subject	(Intercept)	1.29998	1.14017	
Item	(Intercept)	0.49051	0.70036	
Subject	(Intercept)	1.89903	1.37805	
Number of obs: 2353, groups: cond:subj, 78; item, 45; subj, 26				

	**Estimate**	**SE**	***z* value**	**Pr(>|*z*|)**

**Fixed effects**				
(Intercept)	−2.8459	0.4287	−6.639	<0.000***
pu.in	1.6691	0.4515	3.697	<0.000***
pu.apu	1.6193	0.4532	3.573	<0.000***
**C**	**AIC**	**BIC**	**logLik**	**Deviance**
0.8849672	2025	2060	−1007	1.14017

**ACCURACIES IN INCORRECT CONDITIONS OF WORDS WITH CANONICAL PENULTIMATE STRESS (PU.in, PU.apu)**				

**Groups**	**Name**	**Variance**	**SD**	

**Random effects**				
Cond:subject	(Intercept)	1.07975	1.03911	
Item	(Intercept)	0.55879	0.74752	
Subject	(Intercept)	2.34845	1.53247	
Number of obs: 1538, groups: cond:subj, 52; item, 30; subj, 26				

	**Estimate**	**SE**	***z* value**	**Pr(>|*z*|)**

**Fixed effects**				
(Intercept)	−1.18804	0.42685	−2.783	0.00538**
pu.apu	−0.05763	0.42780	−0.135	0.89284
**C**	**AIC**	**BIC**	**logLik**	**Deviance**
0.8693806	1528	1555	−759.1	1518

In order to keep subject and item variation under statistical control, subject, and item were included as random effects. We tested the necessity of these random effects with log-likelihood tests, which always showed that the inclusion of these random effects was justified. In addition, log-likelihood tests revealed that in the best model the random effect of subject is nested within the fixed effect of condition. This indicates that subjects’ responses varied over conditions. Furthermore, inclusion of random contrasts of subject and/or item did not improve the model and were consequenctly discarded.

The models given in Table 4 (in which the correct conditions served as baselines) show significant main effects for both APU.in and APU.pu as well as PU.in and PU.apu indicating that the accuracies for incorrect conditions differ significantly from those of the correct ones. The overall predictive accuracy of the models is very high, with a concordance index of 0.91 for the model of words with antepenultimate stress and 0.89 for the model of words with penultimate stress. The right panels of Figures 2B,D illustrate that the probability of incorrectness based on the final models is higher for incorrect conditions compared to correct conditions, i.e., it is more probable that participants make false judgments in violation conditions than in conditions with correct stress. This is in line with the descriptive means of incorrect responses given in the left Figures 2A,C, which are not corrected for the influence of random variables.

Models calculated over incorrect conditions only (see Table 4) did not reveal significant differences of accuracy between incorrect conditions. The overall predictive accuracy of the models is high, with a concordance index of 0.90 for the model of words with antepenultimate stress and 0.87 for the model of words with penultimate stress.

Taken together, it has been shown that Polish participants have more difficulties to reject the incorrect stress patterns than to accept the correct ones in both word types (with target antepenultimate or penultimate stress). Therefore, we observe that Polish participants have considerable difficulties to reject incorrect stress patterns in general. One could have expected that Polish native speakers have problems to reject only incorrect penultimate stress due to the default status of this stress pattern, but this proved not to be the case.

Reactions to the critical stimuli were not made immediately, but with some timing delay at the offset of the whole sentences, which makes reaction time data not meaningful in this experiment.

### ERP data

**Figure 3:**
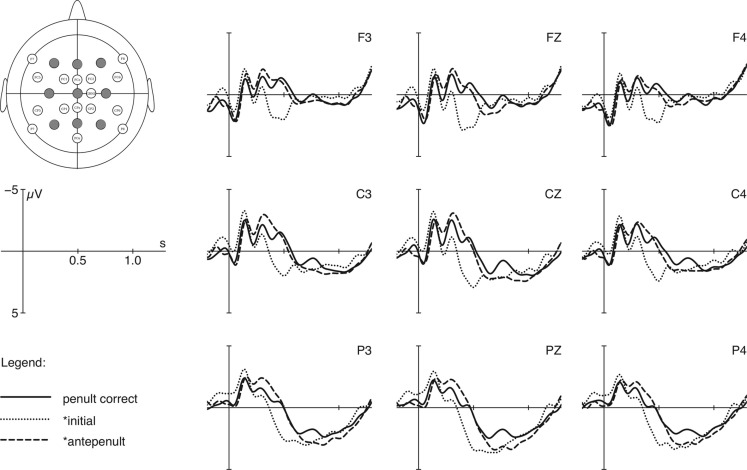
**EEG plots for stimuli with correct penultimate stress (solid line) and with shifts to initial syllable (dotted line) and to antepenultimate syllable (dashed line)**. Electrode cap schemes by Marius’t Hart, permission granted by the author.

**Figure 4:**
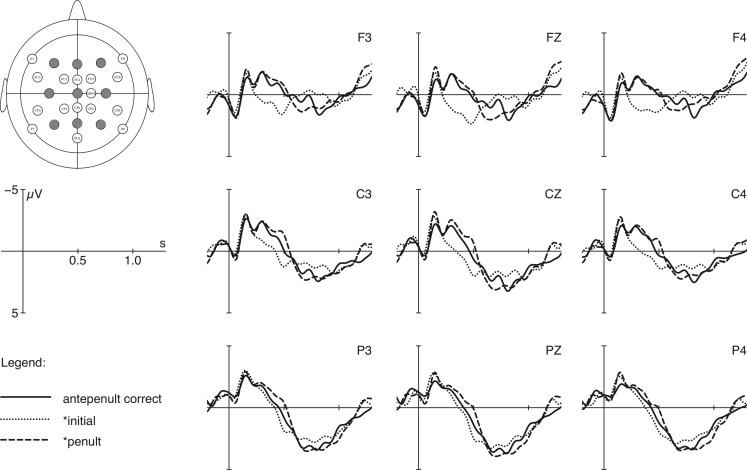
**EEG plots for stimuli with correct antepenultimate stress (solid line) and with shifts to initial syllable (dotted line) and to penultimate syllable (dashed line)**. Time from target onset is plotted on the *x*-axis, and amplitude is plotted on the *y*-axis (negativity upwards).

Grand average ERP curves for the two word types (penultimate vs. antepenultimate stress) with three stress conditions are shown in Figures 3 and 4 respectively. Each of the figures presents 9 electrodes (F3, Fz, F4, C3, Cz, C4, P3, Pz, P4) out of 24. The grand averages of ERPs are plotted from the onset of the critical words up to 1500 ms thereafter with a pre-stimulus baseline of 200 ms. For graphical display only, data were filtered off-line with an 8-Hz low pass filter.

The comparison of the correct condition with each incorrect condition revealed ERP components appearing in different time windows linked to the incorrect stress positions. The selection of time windows in the analysis reported below is based on previous findings (Knaus et al., 2007; Domahs et al., 2008, 2012), with some adjustments being done by visual inspection of grand average curves.

For words with correct penultimate stress (see Figure 3), time windows between 170 and 430 ms, between 210 and 560 ms, and between 620 and 1050 ms were selected. A main effect for the factor stress position was obtained for each comparison. Between 210 and 560 ms, violations with initial stress evoked a significant positivity effect [*F*(1, 25) = 58.62, *p* = 0.000, $\eta_{\text{p}}^{2}$: 0.701], and violations with antepenultimate stress produced a biphasic component: a negativity effect between 170 and 430 ms [*F*(1, 25) = 11.40, *p* = 0.002, $\eta_{\text{p}}^{2}$: 0.313] and a positivity effect between 620 and 1050 ms [*F*(1, 25) = 4.95, *p* = 0.035, $\eta_{\text{p}}^{2}$: 0.165; for a detailed statistical description see Table 5]. Overall, we find pronounced effects produced by incorrect initial and antepenultimate stress.

**Table 5:** **Words with canonical penultimate stress: results of statistical analyses of mean voltage changes for the factor stress position (correct penult vs. incorrect initial and penult) calculated over regions (frontal: F3, Fz, F4; central: C3, Cz, C4; parietal: P3, Pz, P4)**.

COMPARISON BETWEEN CANONICAL PENULTIMATE STRESS AND OTHER STRESS POSITIONS			
**Compared stress positions**	**Time window**	**Polarity**	**Statistics**

Penult vs. initial	210–560 ms	Positivity	Stress position: F(1, 25) = 58.62, *p* = 0.000, $\eta_{\text{p}}^{2}$: 0.701
			Region: *F*(2, 50) = 2.86, *p* = 0.099, $\eta_{\text{p}}^{2}$: 0.103
			Stress position × region: F(2, 50) = 7.76, *p* = 0.007, $\eta_{\text{p}}^{2}$: 0.237
			Stress position in frontal region: *F*(1, 25) = 54.59, *p* = 0.000, $\eta_{\text{p}}^{2}$: 0.686
			Stress position in central region: *F*(1, 25) = 69.56, *p* = 0.000, $\eta_{\text{p}}^{2}$: 0.736
			Stress position in parietal region: *F*(1, 25) = 31.16, *p* = 0.000, $\eta_{\text{p}}^{2}$: 0.555
Penult vs. antepenult	170–430 ms	Negativity	Stress position: F(1, 25) = 11.40, *p* = 0.002, $\eta_{\text{p}}^{2}$: 0.313
			Region: *F*(2, 50) = 10.40, *p* = 0.002, $\eta_{\text{p}}^{2}$: 0.294
			Stress position × region: F(2, 50) = 0.883, *p* = 0.388, $\eta_{\text{p}}^{2}$: 0.034
	620–1050 ms	Positivity	Stress position: F(1, 25) = 4.95, *p* = 0.035, $\eta_{\text{p}}^{2}$: 0.165
			Region: *F*(2, 50) = 24.44, *p* = 0.000, $\eta_{\text{p}}^{2}$: 0.494
			Stress position × region: F(2, 50) = 3.71, *p* = 0.060, $\eta_{\text{p}}^{2}$: 0.129

**p*-Values are Huynh–Feldt-corrected and effect sizes are given as partial eta squared values ($\eta_{p}^{2}$). Significant interactions are resolved and *p*-values of resolved interactions are Bonferroni-corrected*.

For words with correct antepenultimate stress (Figure 4), time windows between 250 and 550 ms and between 600 and 760 ms were selected. Between 250 and 550 ms, repeated measures ANOVAs over the factors stress position (initial, antepenultimate, penultimate) and region (frontal, central, parietal) yield a main effect for the factor stress position (an overview of statistical results is given in Table 6 below). Such an effect occurred as a pronounced positivity evoked by violations with initial stress [*F*(1, 25) = 36.39, *p* = 0.000, $\eta_{\text{p}}^{2}$: 0.593] and as a negativity produced by violations with penultimate stress [*F*(1, 25) = 5.96, *p* = 0.022, $\eta_{\text{p}}^{2}$: 0.193]. For violations involving penultimate stress, the ANOVA calculated over mean voltage changes between 600 and 760 ms showed no main effects [*F*(1, 25) = 2.47, *p* = 0.128, $\eta_{\text{p}}^{2}$: 0.090], but an interaction of the factors stress position and region [*F*(2, 50) = 4.34, *p* = 0.040, $\eta_{\text{p}}^{2}$: 0.148], for which *post hoc* analyses did not reach significance.

**Table 6:** **Words with canonical antepenultimate stress: results of statistical analyses of mean voltage changes for the factor stress position (correct antepenult vs. incorrect initial and penult) calculated over regions (frontal: F3, Fz, F4; central: C3, Cz, C4; parietal: P3, Pz, P4)**.

COMPARISON BETWEEN CANONICAL ANTEPENULTIMATE STRESS AND OTHER STRESS POSITIONS			
**Compared stress positions**	**Time window**	**Polarity**	**Statistics**

Antepenult vs. initial	250–550 ms	Positivity	Stress position: F(1, 25) = 36.39, *p* = 0.000, $\eta_{\text{p}}^{2}$: 0.593
			Region: *F*(2, 50) = 3.26, *p* = 0.075, $\eta_{\text{p}}^{2}$: 0.115
			Stress position × region: F(2, 50) = 13.57, *p* = 0.001, $\eta_{\text{p}}^{2}$: 0.352
			Stress position in frontal region: *F*(1, 25) = 32.20, *p* = 0.000, $\eta_{\text{p}}^{2}$: 0.563
			Stress position in central region: *F*(1, 25) = 36.82, *p* = 0.000, $\eta_{\text{p}}^{2}$: 0.596
			Stress position in parietal region: *F*(1, 25) = 8.99, *p* = 0.018, $\eta_{\text{p}}^{2}$: 0.264
Antepenult vs. penult	250–550 ms	Negativity	Stress position: F(1, 25) = 5.96, *p* = 0.022, $\eta_{\text{p}}^{2}$: 0.193
			Region: *F*(2, 50) = 4.89, *p* = 0.030, $\eta_{\text{p}}^{2}$: 0.164
			Stress position × region: F(2, 50) = 2.21, *x* = 0.145, $\eta_{\text{p}}^{2}$: 0.081
	600–760 ms	Positivity	Stress position: F(1, 25) = 2.47, *p* = 0.128, $\eta_{\text{p}}^{2}$: 0.090
			Region: *F*(2, 50) = 27.88, *p* = 0.000, $\eta_{\text{p}}^{2}$: 0.527
			Stress position × region: F(2, 50) = 4.34, p = 0.040, $\eta_{\text{p}}^{2}$: 0.148
			Stress position in frontal region: *F*(1, 25) = 4.32, *p* = 0.144, $\eta_{\text{p}}^{2}$: 0.147
			Stress position in central region: *F*(1, 25) = 2.40, *p* = 0.401, $\eta_{\text{p}}^{2}$: 0.088
			Stress position in parietal region: *F*(1, 25) < 1, $\eta_{\text{p}}^{2}$: 0.021

**p*-Values are Huynh–Feldt-corrected and effect sizes are given as partial eta squared values ($\eta_{p}^{2}$). Significant interactions are resolved and *p*-values of resolved interactions are Bonferroni-corrected*.

Summarizing, the ERP study revealed asymmetrical results for the three presented stress violations. In comparison to correct conditions with penultimate and antepenultimate stress, violations involving initial stress produce enhanced positivity effects between 200 and 500 ms post word onset. Secondly, violations involving antepenultimate stress, when penultimate stress is the target, produce a biphasic ERP component: a broadly distributed negativity between 200 and 400 ms followed by an enhanced positivity between 600 and 1000 ms. In contrast, violations with penultimate stress in the context of antepenultimate target reveals a negativity between 250 and 550 ms, but no positivity effect at all. These results are summarized in Table 7 in which the time windows for significant effects are given.

**Table 7:** **Significant effects (presented as time windows) for types of stress shifts**.

	APU → PU	APU → IN	PU → APU	PU → IN
Negativity	250–550 ms	–	170–430 ms	–
Positivity	–	250–550 ms	620–1050 ms	210–560 ms

As noted earlier, ERP effects have been found to occur for many conditions although participants displayed rather low accuracy scores in the rejection of items bearing incorrect stress (see Table 4). This raises the question whether the same ERP effects are found when only trials with correct responses are considered. For this purpose, we analyzed the ERP data of those trials for which correct judgments had been collected (72% of all trials) using the same time windows and factors. The results matched those obtained for the analyses on the whole set of data (with correct and incorrect judgments), except for the fact that the negativity between 250 and 550 ms for incorrect penultimate stress in the condition of correct antepenultimate stress did not reach significance any more [with *F*(1, 25) = 2.92, *p* < 0.1].

## Discussion

In the current experiment, native speakers of Polish were confronted with correctly and incorrectly stressed words of their language in order to investigate (i) whether default stress patterns are processed differently from exceptional or post-lexical stress patterns, and (ii) whether metrical structure in terms of feet constrains processing. The participants’ task was to judge explicitly the correctness of each stress pattern. This evaluation was carried out on the basis of the expectation of a specific stress pattern that was anticipated by the visual presentation of each critical item before it was presented auditorily with correct or incorrect stress.

### Behavioral data

In the stress evaluation task, error rates ranged between 32 and 44% for incorrect conditions disregarding whether the default (PU) or non-default stress (APU and IN) was involved. The accuracy scores obtained for incorrect conditions per word type did not differ. Thus, one possible hypothesis that the default stress pattern can be over-applied to lexical stress conditions, but not vice versa, is not supported by the behavioral data. The data suggest that Polish speakers are not very confident in their judgment of stress violations when processing stress information at the conscious level. However, responses to different incorrect conditions differ when taking the ERP results into consideration. Furthermore, the comparable accuracy in responding to different types of incorrect forms do not allow an answer to the question whether foot structure affects metrical processing.

### ERP data for words with canonical default stress

Violations of words with canonical default stress (e.g., *atmo*′*sfera*) revealed (i) a positivity between 210 and 560 ms for initial stress and (ii) a biphasic pattern for antepenultimate stress: a negativity between 170 and 430 ms and a positivity between 620 and 1050 ms. For the early and very pronounced positivity effect obtained for shifts to the initial syllable, different interpretations are at hand. Firstly, the positivity effect can be interpreted as belonging to the P3b family to reflect that the language processor encounters a deviant strong syllable very early in the auditory stimulus, and that the evaluation process is finished even before lexical processing steps are initiated. Due to the visual presentation of the words before the auditory presentation, participants were expected to hear words with penultimate stress instead of initial stress. Such positivity effects linked to the evaluation of prosodic or rhythmical deviations have also been reported by, for instance, Brochard et al. (2003), Domahs et al. (2008), Knaus and Domahs (2009), or Magne et al. (2007). A similar positivity effect is also observed for illegal non-word stimuli presented in lexical decision tasks (e.g., Holcomb and Neville, 1990; Bentin et al., 1999). In these studies, illegal non-words were (in contrast to legal pseudowords) categorized as non-existent in a particular language even before processes of lexical retrieval were initiated. In the core phonological system of Polish, initial stress may also be considered as an outside or illegal pattern, leading to rather early closure of phonological processing and categorization, which is reflected by a P3b.

Alternatively, positivity effects in metrical tasks have also been interpreted as instances of P600 effects (e.g., Marie et al., 2011; Schmidt-Kassow et al., 2011a,b). In two experiments in which morphosyntactic violations and/or metrical inhomogeneity had to be judged (Schmidt-Kassow et al., 2011b), syntactically as well as metrically incorrect conditions evoked a positivity between 700 and 1100 ms post stimulus onset. Thus, positivity effects in evaluation tasks are differently labeled, although they seem to reflect quite comparable processes, namely the sensitivity to a deviant structure with an amplitude being correlated with the degree of abnormality (e.g., Picton, 1992; Coulson et al., 1998). For example, the less likely a metrical structure the more pronounced the positivity effect. Regardless, whether a P300 or a P600, both effects reflect a sensitivity to some deviant structure.

The electrophysiological responses to incorrect initial stress, i.e., the occurrences of positivity effects, are furthermore not in line with the assumption that quadrisyllabic words with penultimate stress consist of two trochees [as in *(*_′_*at.mo)_Fw_(*′*sfe.ra)_Fs_*]. According to such a metrical analysis (Dogil and Williams, 1999), the initial syllable is the locus of secondary stress, and, thus, potentially a good landing site for shifted stresses in experimental conditions with manipulated word stress. As evidence for this structure and in analogy with the results by Domahs et al. (2008), it was expected that initial stress would not evoke a positivity effect in Polish. Taking into account that dialects allow for initial stress and that the occurrence of initial stress is pragmatically licensed, the positivity effect found is an unexpected result which calls for further investigations on the role of initial stress in future research.

Violations involving antepenultimate stress produced a biphasic ERP component: a negativity between 170 and 430 ms and a positivity between 620 and 1050 ms. For the negativity effect, two potential interpretations should be considered. One is that the negativity belongs to the N400 family and reflects enhanced costs in lexical retrieval which has also been found in Knaus et al. (2007) for ill-stressed German words (without visual presentation prior to the auditory stimuli) and for violations of lexically specified stresses in Turkish (Domahs et al., 2012). In the present experiment, however, neither were the stimuli unknown prior to auditory presentation nor do we assume that violations of words with canonical penultimate stress are processed as lexical violations. We therefore favor the second interpretation, according to which this negativity effect (whose onset starts earlier compared to the classical N400 time window between 300 and 500 ms) reflects a generalized error-detection mechanism (e.g., Koelsch et al., 2000; Brochard et al., 2003; Abecasis et al., 2005; Rothermich et al., 2010; Schmidt-Kassow et al., 2011a). For example, such a negativity effect has been found between 200 and 350 ms for deviations of metrical regularity (an alternating sequence of strong and weak syllables) in jabberwocky sentences (Rothermich et al., 2010) and also for violations of sound expectancy during the processing of music between 190 and 250 ms. The latter effect has been labeled ERAN (early right anterior negativity) due to a pronounced distribution in anterior frontal region (e.g., Koelsch et al., 2000). Analogously, our suggestion is that the present negativity reflects the violation of the expectancy to encounter a particular rhythmical pattern, namely penultimate stress. In the present experimental design, this expectation is triggered by the visual presentation of each critical stimulus before auditory stimulus presentation. Thus, the main difference between the Turkish and the Polish study is that in Turkish a lexical expectation was violated, while in Polish a rhythmical expectation was not met.

The pronounced positivity effect around 620–1050 ms for incorrect antepenultimate stress is interpreted in line with Domahs et al. (2008) and Domahs et al. (2012) to reflect an evaluation process of a prosodic mismatch between the expected correct and encountered incorrect stress patterns. In other words, a shift to the antepenultimate position resulted in a deflection that is claimed to correspond to the ease with which participants rejected an incorrect input. This finding suggests that Polish participants are sensitive to this exceptional stress pattern. However, once again it is not expected that Polish participants would produce 32% of misjudgments when confronted with incorrect antepenultimate stress because the ERP response indicates that this stress pattern is processed as deviation from the expectation.

As regards our hypotheses related to foot structure, the effect patterns found do not support the assumption that foot structure plays a role in the processing of ill-stressed forms. The crucial test cases are the shifts from penultimate to initial syllables [*(′*at.mo*)*_Fw_*(_′_*sfe.ra*)*_Fs_*] which should have led to either a reduced P300 effect or none at all, but did evoke a pronounced one.

### ERP data for words with canonical antepenultimate stress

Violations for words with exceptional antepenultimate stress produced the following results. First of all, stress shifts to initial syllables again evoked a positivity effect around 250 and 550 ms and therefore indicate that initial stress is not a potential stress position in such words.

Second, for the shift from antepenultimate to penultimate syllable, a negativity effect between 250 and 550 ms was obtained, but no positivity effect. With respect to this negativity, a similar latency and distribution was found as in the mirror-inverted case mentioned above. Likewise, we interpret this effect as an instance of an error-detection component reflecting the processing of an unexpected stress pattern. However, in the comparable study performed with Turkish participants (Domahs et al., 2012) the over-application of the default stress to words displaying a lexicalized stress pattern yielded an N400-like effect rather than an error-detection component. It is noteworthy here that in Turkish the default stress is no acceptable variant for words with lexical stress. Thus, lexical specifications are violated when lexical stress is shifted to the default position. This is not the case in Polish: shifts to the penult for words with antepenultimate stress are possible pronunciation variants. This observation together with the latency of the negativity suggests that the present negative deflection reflects the detection of an unexpected stress pattern that does not necessarily violate the lexical specification for antepenultimate stress. Furthermore, it must be emphasized that the occurrence of this negativity effect is less stable than the other effects as it is no longer significant when only correct answers are included in the ERP analysis. The error-rate for this condition is the highest and, therefore, 44% of trials are discarded from the analysis.

Finally, in contrast to other stress violations presented in the experiment, violations involving penultimate stress did not evoke a P300 effect. This lack of an effect is in line with the processing of incorrect default stress in Turkish showing that violations involving the default pattern are harder to evaluate and to classify as incorrect compared to other violations. The ERP patterns found in the present experiment, thus, reflect the basic distinctions of the Polish word stress system, which treats penultimate stress differently from both antepenultimate and initial stress, even if these distinctions are not visible in the behavioral data.

To summarize, according to the hypotheses put forward in this paper, our conclusions are the following: the processing differences found for different stress violations can best be described along the default/non-default distinction. These observations lead us to the conclusion that Polish speakers are only partially stress “deaf.” In particular, our data show that the inability to evaluate stress is restricted to the default. We may therefore speculate that Polish speakers have difficulties to distinguish the default pattern from other stress patterns in newly learned words, since the default is processed less consciously than other patterns. Such difficulties do not arise when comparing non-default patterns. For the behavioral data, we conclude that Polish speakers have severe problems in meta-linguistic judgments on incorrect word stress.

Overall, the effect patterns found are not modulated by metrical properties ascribed to Polish. For this reason, the present data cannot support metrical analyses of Polish that assume a parsing of syllables into feet throughout the prosodic word, i.e., quadrisyllabic words consisting of two bisyllabic trochees. Whether Polish prosodic words end in a trochee can neither be supported nor challenged by the present results.

## Conflict of Interest Statement

The authors declare that the research was conducted in the absence of any commercial or financial relationships that could be construed as a potential conflict of interest.

## Appendix

**Table:**

Words with penultimate stress	Words with antepenultimate stress
Atmosfera	Balistyka
Bateryjka	Curriculum
Cukiernica	Egzotyka
Czarownica	Erotyka
Dyspozycja	Fortissimo
Eksperyment	Genezaret
Elementarz	Gimnastyka
Estradowiec	Jeruzalem
Kalifornia	Johannesburg
Karaiby	Lingwistyka
Monotonia	Los Angeles
Przedszkolanka	Mount Everest
Psychologia	Polityka
Rodzicielstwo	Republika
Technologia	Turystyka
